# Cellulose–Amine Porous Materials: The Effect of Activation Method on Structure, Textural Properties, CO_2_ Capture, and Recyclability

**DOI:** 10.3390/molecules29051158

**Published:** 2024-03-05

**Authors:** Sarah Krupšová, Miroslav Almáši

**Affiliations:** 1 Novy PORG Gymnasium, Pod Krcskym lesem 25, CZ-142 00 Prague, Czech Republic; krupsovas@gmail.com; 2 Department of Inorganic Chemistry, Faculty of Science, Pavol Jozef Safarik University, Moyzesova 11, SK-040 01 Kosice, Slovakia

**Keywords:** cellulose, amines, hydrothermal synthesis, pyrolysis, carbon dioxide storage

## Abstract

CO_2_ capture via physical adsorption on activated porous carbons represents a promising solution towards effective carbon emission mitigation. Additionally, production costs can be further decreased by utilising biomass as the main precursor and applying energy-efficient activation. In this work, we developed novel cellulose-based activated carbons modified with amines (diethylenetriamine (DETA), 1,2-bis(3-aminopropylamino)ethane (BAPE), and melamine (MELA)) with different numbers of nitrogen atoms as in situ *N*-doping precursors. We investigated the effect of hydrothermal and thermal activation on the development of their physicochemical properties, which significantly influence the resulting CO_2_ adsorption capacity. This process entailed an initial hydrothermal activation of biomass precursor and amines at 240 °C, resulting in **C+DETA**, **C+BAPE** and **C+MELA** materials. Thermal samples (**C+DETA (P)**, **C+BAPE (P)**, and **C+MELA (P)**) were synthesised from hydrothermal materials by subsequent KOH chemical activation and pyrolysis in an inert argon atmosphere. Their chemical and structural properties were characterised using elemental analysis (CHN), infrared spectroscopy (IR), scanning electron microscopy (SEM), and thermogravimetric analysis (TG). The calculated specific surface areas (*S_BET_*) for thermal products showed higher values (998 m^2^ g^−1^ for **C+DETA (P)**, 1076 m^2^ g^−1^ for **C+BAPE (P)**, and 1348 m^2^ g^−1^ for **C+MELA (P)**) compared to the hydrothermal products (769 m^2^ g^−1^ for **C+DETA**, 833 m^2^ g^−1^ for **C+BAPE**, and 1079 m^2^ g^−1^ for **C+MELA**). Carbon dioxide adsorption as measured by volumetric and gravimetric methods at 0 and 25 °C, respectively, showed the opposite trend, which can be attributed to the reduced content of primary adsorption sites in the form of amine groups in thermal products. N_2_ and CO_2_ adsorption measurements were carried out on hydrothermal (**C**) and pyrolysed cellulose (**C (P)**), which showed a several-fold reduction in adsorption properties compared to amine-modified materials. The recyclability of **C+MELA**, which showed the highest CO_2_ adsorption capacity (7.34 mmol g^−1^), was studied using argon purging and thermal regeneration over five adsorption/desorption cycles.

## 1. Introduction

The rapidly rising production of anthropogenic carbon dioxide has been a major factor related to global climate change for the last few decades. CO_2_, which accounts for 75% of total greenhouse gases (GHGs) [1], has experienced an increase in concentrations throughout the entire timeframe, with the current concentration surpassing 421 ppm [2]. This has resulted in the production of irreversible changes to the planet’s biosphere, such as global warming, rising temperatures, acidification of oceans, and more frequent occurrences of extreme weather events, which are responsible for alterations in water resource availability [3], decreased biodiversity due to extinction of species [4], and forced displacements of populations due to climate-driven externalities [5]. All of this significantly exacerbates the quality of life in both developing and developed countries [6,7,8]. Therefore, the effective mitigation of atmospheric carbon dioxide has become the main focus of attention.

Post-combustion capture of CO_2_ (PCC) from flue gas has potential for mitigating carbon emissions. The process consists of separating carbon dioxide from the emissions produced during the combustion of fossil fuels [9]. In order to achieve extraction, liquid or solid sorbents with high affinity to CO_2_ molecules are utilised [10,11]. In general, the CO_2_ capture step of PCC technology accounts for approximately 70% of the system’s total cost [12]. Thus, the selection of an effective sorbent with a low production price is of vital importance. However, currently applied PCC technology is not economically viable due to the use of amine scrubbing, which suffers from low recyclability [13], as well as to energy-inefficient operation due to the presence of water, which possesses high specific heat capacity and contributes to the corrosion of vessels [14].

Therefore, developing efficient, ecological, and low-cost CO_2_ capture materials is urgently required. Porous materials such as activated carbons [15,16], carbon nanotubes [17,18], covalent–organic frameworks [19,20], metal–organic frameworks [21,22,23,24], zeolites [25,26,27], mesoporous silicas [28,29,30], and natural materials based on nanoporous shales, limestones, and foams [31,32,33,34] stand out as promising frontiers in the quest for effective carbon capture and storage technologies. Recently, sorbents based on activated porous carbons (APC) have received increased interest because of their unique physio-chemical properties, such as high surface area [35,36], tunable pore distribution [37], and the possibility of chemical modification for enhanced affinity to CO_2_ molecules [38,39,40]. Additionally, their low isosteric heat of adsorption allows for effective regeneration after CO_2_ adsorption and enables them to resist moisture, making APCs more economically and energetically feasible in industrial CO_2_ capture. Furthermore, their production cost can be further decreased by utilising biomass as the primary precursor. Chemical or physical activations are frequently used methods to develop effective APCs with high porosity. Hence, our research aimed to develop novel biomass-based activated carbons with low production cost and investigate the effect of hydrothermal and thermal activation methods on the development of their textural properties, which largely influence the material’s CO_2_ adsorption capacity.

Amine modification of porous materials is a widely explored strategy to enhance carbon dioxide storage capacity, particularly in the context of carbon capture and storage technologies. Amine functionalisation involves introducing amine groups (-NH_2_) onto the surface of porous materials. This modification imparts specific chemical interactions with CO_2_ molecules, leading to increased adsorption capacity [41,42]. Various types of carbonaceous materials with a high CO_2_ storage capacity can be listed, such as nitrogen-doped microporous carbons containing the benzoxazine moieties PAEBZ-A and PAMBZ-A (*S_BET_* = 960 and 464 m^2^ g^−1^) [43], amine-modified graphene oxide (*S_BET_* = 1772 m^2^ g^−1^) [44], *N*-doped carbon derived from polypyrrole (*S_BET_* = 1521 m^2^ g^−1^) [45], and biomass-derived carbon-based material from date waste (*S_BET_* = 2367 m^2^ g^−1^) [46], with CO_2_ adsorption capacities of 31.69 wt. %, 20.24 wt. %, 8.90 wt. %, 7.16 wt. %, and 6.40 wt. %, respectively, at 0 °C and 1 bar.

**Figure 1:**
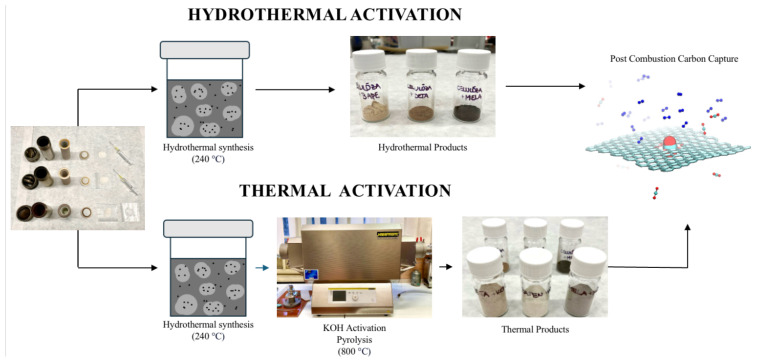
The preparation procedure of cellulose–amine porous materials using hydrothermal and thermal activation.

Herein, APCs samples based on cellulose as the primary precursor doped with *N*-containing modifiers (diethylenetriamine (DETA), 1,2-bis(3-aminopropylamino)ethane (BAPE), and melamine (MELA)) were synthesised via facile single-step hydrothermal and two-step hydrothermal-thermal activation methods (see Figure 1). The prepared APCs were entitled as C+N, where N refers to the type of amine used for *N*-doping modification and C is carbon. Additionally, activated carbons prepared from the same precursor and *N*-doping modifier but through a different activation method are differentiated by the symbol (P), denoting the involvement of pyrolysis in the material’s synthesis. The compositions, structures, and CO_2_ capture properties of all the materials were studied by CHN elemental analysis, infrared spectroscopy (IR), thermogravimetric analysis (TG), scanning electron microscopy (SEM), nitrogen adsorption, and CO_2_ adsorption, while the recyclability was studied by temperature/gas purging-programmed CO_2_ adsorption. It has been observed that the degree of porosity, specific surface area, and amine content can be well-tuned by selection of the activation method. In contrast, the chemical composition and CO_2_ capture ability depend vastly on the chemical structure; hence, *N*-doping modifiers are applied in the synthesis. **C+MELA (P)** showed superior pore volume and *S_BET_* surface area, equaling 0.628 cm^3^ g^−1^ and 1348 m^2^ g^−1^, respectively. However, the most effective material for CO_2_ capture under varying temperatures (0 and 25 °C) was **C+MELA**, which attained the highest CO_2_ adsorption capacity of 7.34 mmol g^−1^ at 0 °C and 1 bar and 4.64 mmol g^−1^ at 25 °C and 1 bar.

## 2. Results and Discussion

### 2.1. Composition and Structural Characterisation

The elemental composition of the prepared APCs in wt. % is summarised in Table 1. In hydrothermal samples, the nitrogen content is in the range of 6.5–12.6 wt. % and increases depending on the nitrogen content in the amine used (DETA = 3 × N, BAPE = 4 × N, and MELA = 6 × N). As indicated by the data, pyrolysis of the samples (P) led to significant enhancement of the carbon matrix as well as to a considerable decrease of the wt. % composition of hydrogen, nitrogen, and oxygen in the structures. In particular, the reduction in N content suggests a significant loss of amine functional groups during the pyrolysis step. This can be attributed mainly to the high activation temperature applied during pyrolysis, which led to the release of small inorganic molecules (NH_3_, H_2_O, CO_2_, CO) from the structure of porous materials.

**Figure 2:**
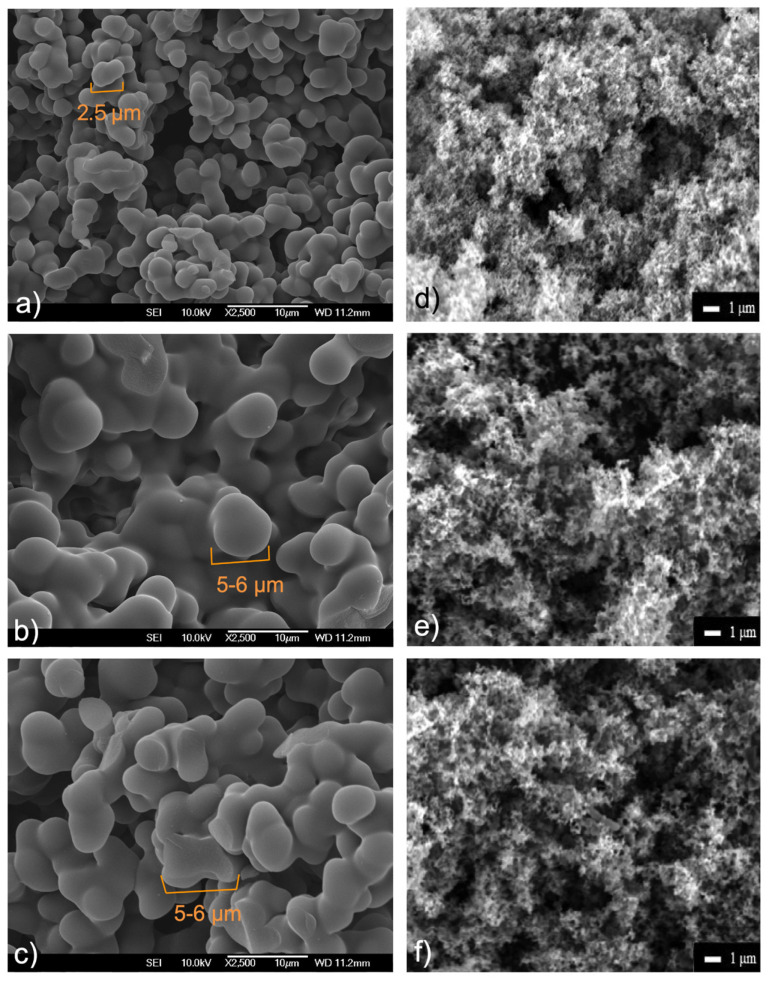
SEM images of hydrothermal: (**a**) **C+MELA**, (**b**) **C+BAPE**, (**c**) **C+DETA** and thermal: (**d**) **C+MELA (P)**, (**e**) **C+BAPE (P)** (**f**) **C+DETA (P)** products (structures of APCs modified with the same amine are shown beside each other).

SEM images (Figure 2) with magnification 2500× were used to evaluate the morphologies of the prepared samples. The hydrothermal products (see Figure 2a–c) display regular spherical morphologies with smooth surfaces, which are directly linked to the particle size (~2.5 μm for **C+MELA**, ~5–6 μm for **C+BAPE**, and ~5–6 μm for **C+DETA**) and shape of the used amines. As seen in Figure 2d–f, thermal activation led to irregular fibrous structures with smaller particle sizes, which can be attributed to both the activating agent and the thermal treatment. While KOH activation led to the development of porosity via pitting and channelling [47], high thermal activation resulted in the loss of volatile matter, causing the formation of voids, which significantly enhanced the degree of porosity and created a well-developed porous matrix observable in the thermal samples.

### 2.2. Spectroscopic Analysis

**Figure 3:**
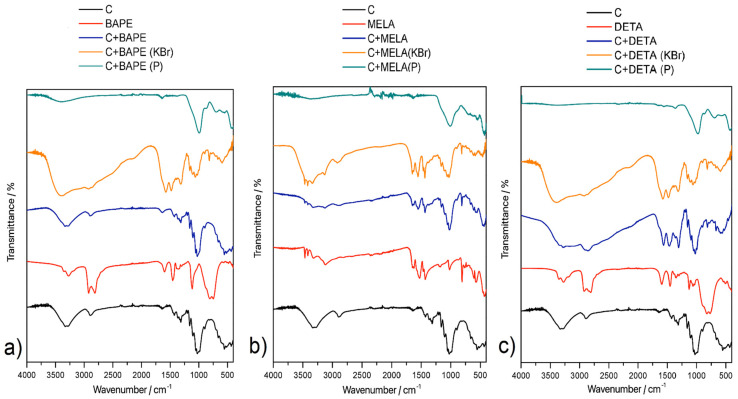
IR spectra of the prepared APCs doped with (**a**) 1,2-bis(triaminopropylamino)ethane (BAPE), (**b**) melamine (MELA), and (**c**) diethylenetriamine (DETA) (black curve—cellulose (C); red curve—corresponding pure amine; blue curve—hydrothermal product C+amine measured by ATR technique; orange curve—hydrothermal product C+amine measured by KBr technique; teal curve—pyrolysed product C+amine (P) measured by ATR technique).

The presence of cellulose and amines in the hydrothermal products was identified by infrared spectroscopy (IR). The IR spectra of the compounds at ambient temperature are shown in Figure 3. The list of the characteristic adsorption bands with corresponding individual vibrations, together with the specific wavenumber values, is provided in Table 2.

In all prepared materials, the successful binding of amine to cellulose can be observed in the form of adsorption bands corresponding to ν(NH) vibrations with values of 3328 cm^−1^ for **C+MELA**, 3184 cm^−1^ for **C+DETA**, and 3180 cm^−1^ for **C+BAPE**. The reaction between the main precursor and amines was confirmed by vibrations corresponding to ν(C-O) of cellulose at 1052 cm^−1^, 1022 cm^−1^ (**C+MELA**), 1062 cm^−1^, 1036 cm^−1^ (**C+DETA**), and 1055 cm^−1^, 1031 cm^−1^ (**C+BAPE**). In all materials, the presence of wavenumbers in the ranges of 2876–2962 cm^−1^ and 2812–2898 cm^−1^ was observed, which respectively correspond to asymmetric and symmetric ν(CH_2_)_aliph_ vibrations and confirm the presence of cellulose in hydrothermal materials. In the IR spectrum of **C+MELA**, the wavenumbers for ν(NH) shifted to lower values, i.e., 3184 cm^−1^ and 3130 cm^−1^, indicating the formation of intermolecular hydrogen bonds between melamine molecules.

Characteristic absorption bands belonging to amine functional groups were not observed in the IR spectra of the pyrolysed materials (see Figure 3 and Table 2), which is in good agreement with the results of the CHN analysis showing N content below 1 wt. % in all thermal products. Wavenumbers of 991 cm^−1^ (**C+MELA (P)**), 977 cm^−1^ (**C+BAPE (P)**), and 992 cm^−1^ (**C+DETA (P)**) were observed in the spectra of the pyrolysed materials, suggesting the presence of deformation γ(CCH) vibrations.

**Figure 4:**
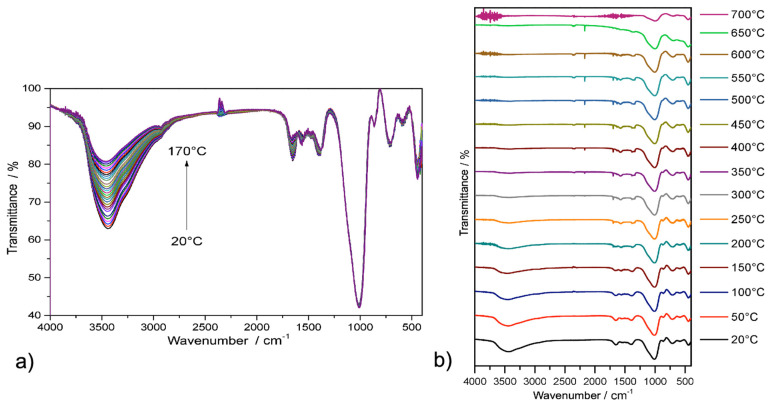
IR spectra of **C+DETA** after gradual heating in the temperature range of (**a**) 20–170 °C with an increment of 5 °C and (**b**) 20–700 °C with an increment of 50 °C.

Infrared spectroscopy with gradual heating in the temperature range of 20–170 °C (see Figure 4a) with 5 °C increment (in situ measurements) and 20–700 °C with an increment of 50 °C (step-by-step heating in an oven; see Figure 4b) was used to investigate the thermal stability of the **C+DETA** sample. Initially, the desolvation process of water in the compound occurred, as indicated by a change in the intensity of ν(OH) vibration located at ~3500 cm^−1^ that decreased with increasing temperature (see Figure 4a). From Figure 4b, it is possible to observe the complete release of water upon heating to 250 °C, when the broad absorption ν(OH) band can no longer be observed. At 350 °C, a significant decrease in the wavenumbers of ν(NH_2_) and ν(NH) was recorded, indicating the decomposition of diethylenetriamine (DETA). Subsequently, a gradual decrease in the wavenumbers of ν(C-O) vibrations was observed in the temperature range of 350–700 °C, proving the release of cellulose. Therefore, the thermal decomposition of the prepared materials proceeds first by dehydration, followed by the decomposition of the amine, and finally by the release of cellulose.

### 2.3. Thermal Stability

**Figure 5:**
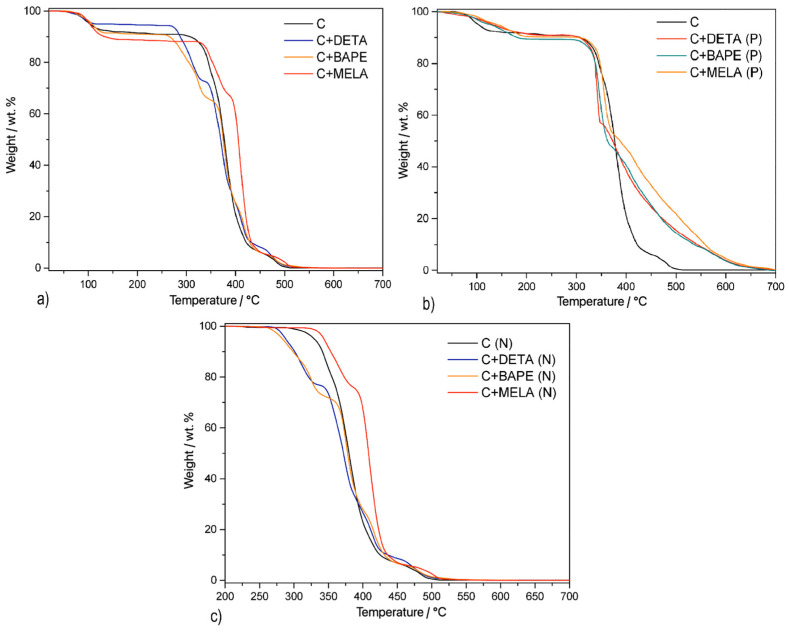
TG curves of the APCs prepared via (**a**) hydrothermal activation, (**b**) thermal activation, and (**c**) normalised TG curves.

The thermal stability of the prepared compounds was investigated by thermogravimetric analysis (TG) in the temperature range of 20–700 °C. Thermoanalytical curves measured in an air atmosphere are shown in Figure 5, and data on the thermal stability of compounds, stability after dehydration, and mass loss in individual temperature intervals are summarised in Table S1 in Supplementary Materials. after subtraction of mass changes corresponding to solvents, normalised TG curves (see Figure 5c) [48] were used to calculate the empirical formulas of the prepared materials and thereby determine the amount of bound amine in hydrothermal samples.

As indicated in Figure 5a, TG curves of hydrothermal samples consist of three notable mass decreases, which, based on the results from IR spectroscopy with gradual heating (see Section 2.2), can be attributed to water, amine, and cellulose, respectively. **C+DETA** showed thermal stability up to 43.5 °C, after which a water loss of 5.0 wt. % occurred. In the temperature range of 115.2–265.3 °C, no significant thermal decomposition or mass decrease was observed, indicating the stability of the material after dehydration. Another mass decrease of 22.1 wt. % was recorded in the temperature range of 265.3–332.3 °C, which can be attributed to the release of diethylenetriamine. In the last phase of the thermal decomposition (332.3–508.9 °C) the mass change was 72.9 wt. %, which can be attributed to the decomposition of cellulose in the material. The empirical formula for **C+DETA** was calculated and identified as C_1_DETA_1_.

Material **C+BAPE** displayed the highest thermal stability up to a temperature of 53.8 °C, after which a mass decrease to 8.5 wt. % was recorded. This weight change corresponded to the release of water, after which the material acquired a stable dehydrated state up to a temperature of 134.1 °C, when the release of 1,2-bis(3-aminopropylamino)ethane was observed in the form of a mass loss of 25.4 wt. %. In the last temperature interval of 341.4–542.2 °C the decomposition of cellulose was observed, causing a decrease in mass of 66.1 wt. %. Thus, the empirical formula of the **C+BAPE** compound was determined to be C_1_BAPE_0.76_.

As seen in Table S1 in Supplementary Materials, **C+MELA** showcased thermal stability up to 39.9 °C, followed by the decomposition of water by 11.1 wt. % up to 163.3 °C. The thermal decomposition of melamine occurred in the second temperature interval (163.3–315.7 °C), as indicated by a mass decrease of 20.4 wt. %. Subsequently, a mass change of 68.5 wt. % was determined in the third temperature interval (380.1–517.4 °C), which can be assigned to the final decomposition of cellulose. Based on the aforementioned mass losses, the empirical formula of the **C+MELA** compound was computed to be C_1_MELA_0.83_.

TG curves for the thermal samples are shown in Figure 5b. As indicated in Table S1 in Supplementary Materials, the decomposition of the thermal compounds initially proceeded by dehydration of the products in the first step, which was accompanied by weight losses of 9.1 wt. %, 10.7 wt. %, and 8.2 wt. % for **C+DETA (P)**, **C+BAPE (P)**, and **C+MELA (P)**, respectively. Subsequently, the dehydrated compounds acquired thermal stability up to a temperature of 283.5 °C for **C+DETA (P)**, 291.6 °C for **C+BAPE (P)**, and 300.0 °C for **C+MELA (P)**. The decomposition of the porous structures of the materials occurred in two mass losses in a wider temperature interval up to 700 °C, which is higher than in the case of hydrothermal products. However, due to the undefined composition of the materials caused by the further development of the carbon matrix during pyrolysis (see Section 3.3), it was not possible to assign individual weight losses clearly.

### 2.4. Textural Properties

In order to evaluate the porosity and textural properties of the prepared APCs, N_2_ adsorption/desorption measurements were carried out at −196 °C and in a relative pressure range of 0–1. The values for specific surface areas were determined by the BET method, and pore volumes were calculated using NLDFT.

**Figure 6:**
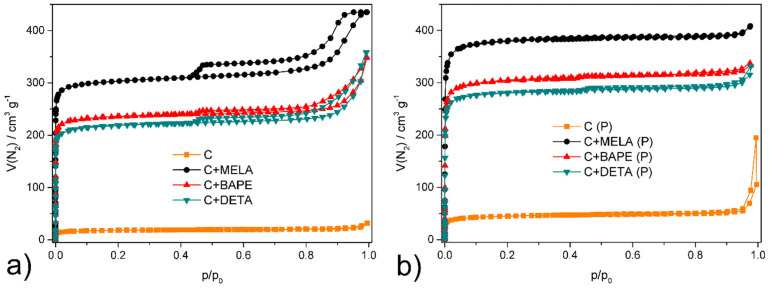
N_2_ adsorption/desorption isotherms as measured at −196 °C for (**a**) hydrothermal and (**b**) thermal products activated at 150 °C under vacuum.

As seen in Figure 6a, N_2_ adsorption/desorption isotherms for hydrothermal products can be classified as type *I* with H5 hysteresis, which indicates reversible micropore filling followed by multilayer physisorption [49]. These observations suggest that the adsorption of hydrothermal products proceeds predominantly on the non-microporous part of the sorbent. As indicated in Figure 6b, N_2_ isotherms for thermal products can be classified as type *I*, with H4 hysteresis caused by capillary condensation of nitrogen in mesopores [50]. Such loops are characteristic of chemically activated carbonaceous materials and suggest well-developed micro- and mesoporosity of the APCs prepared via thermal activation [51,52].

**Figure 7:**
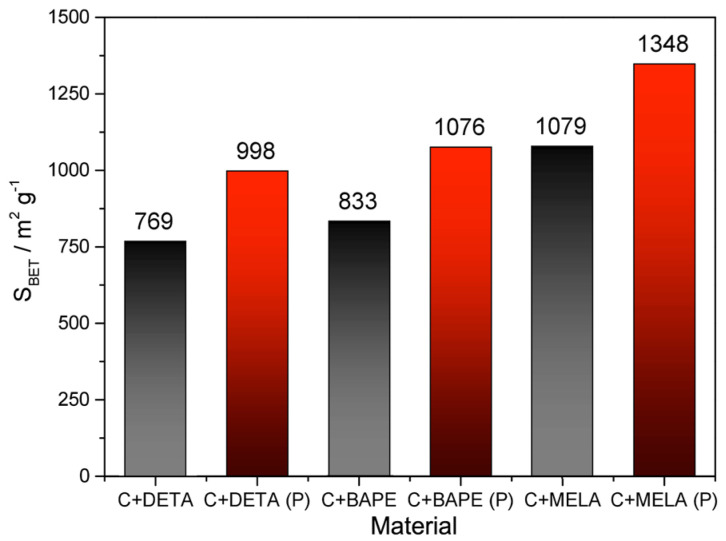
Comparison of *S_BET_* surface areas for hydrothermal (black columns) and thermal (red columns) samples.

In terms of BET surface areas (*S_BET_*) and pore volumes (*V_p_*, see Table 3), more enhanced degrees of porosity were observed on the thermal samples, with the highest values of 1348 m^2^ g^−1^ and 0.628 cm^3^ g^−1^ being reported on melamine-containing material, followed by samples doped with 1,2-bis(3-aminopropylamino)ethane (1076 m^2^ g^−1^; 0.502 cm^3^ g^−1^) and diethylenetriamine (998 m^2^ g^−1^; 0.488 cm^3^ g^−1^). This can be attributed mainly to KOH chemical activation promoting the formation of micro- and mesoporous-activated carbon [53]. The hydrothermal samples displayed well-developed porosity as well, with smaller surface areas compared to the pyrolysed products. The most significant values were observed on melamine APC (1079 m^2^ g^−1^; 0.511 cm^3^ g^−1^), followed by 1,2-bis(3-aminopropylamino)ethane (833 m^2^ g^−1^; 0.473 cm^3^ g^−1^) and diethylenetriamine (769 m^2^ g^−1^; 0.378 cm^3^ g^−1^). Therefore, it can be concluded that, in terms of textural properties, thermal activation led to a significant enhancement of specific surface area values, as seen in Figure 7 and Table 3.

For comparison, hydrothermal (**C**) and thermal (**C (P)**) cellulose were prepared without the addition of amine under the same synthetic conditions. As can be seen from Figure 6, the prepared compounds adsorbed only small amounts of nitrogen, and the determined surface areas of 32 m^2^ g^−1^ for **C** and 121 m^2^ g^−1^ for **C (P)** are several times lower than for the amine-modified materials. The obtained values of *S_BET_* for both materials are comparable with the cellulosic materials reported in [54]. The described results confirm that amines effectively cross-link the polymeric chains of cellulose during synthesis, which leads to the formation of materials with more developed porosity and better textural properties (see comparison in Table 3).

### 2.5. CO_2_ Adsorption

**Figure 8:**
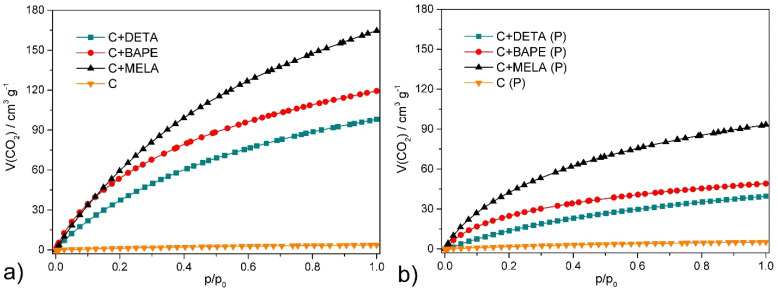
CO_2_ adsorption/desorption isotherms of (**a**) hydrothermal and (**b**) thermal materials measured at 0 °C and up to 1 bar.

All prepared APCs were subjected to CO_2_ adsorption at 0 °C at 1 bar. The values of adsorbed carbon dioxide volume in cm^3^ g^−1^, amount of adsorbed CO_2_ in mmol g^−1^, and weight percent in wt. % are summarised in Table 4. The corresponding CO_2_ adsorption isotherms of the hydrothermal and thermal products are depicted in Figure 8. The isotherms were identified as type *I* according to the IUPAC classification [55]. As indicated in Figure 8, the CO_2_ uptake generally increases at higher adsorption pressure. Despite lower values of specific surface area and pore volume, the hydrothermal products displayed higher adsorption capacity, which suggests a nonlinear relationship between BET surface area and adsorption capacity for the prepared biomass-based activated carbons. The high CO_2_ uptake of the hydrothermal products can be attributed to their high amine (nitrogen) content, as indicated by CHN/O analysis (see Table 1), which serve as the main adsorption sites for CO_2_ molecules [56,57]. The highest adsorption capacity of 32.3 wt. % was recorded on melamine-containing hydrothermal APC (**C+MELA**), which represents one of the highest CO_2_ uptakes measured on biomass-derived APCs yet reported in the literature (see Table 5 for comparison). High respective adsorption capacities of 23.46 wt. % and 19.28 wt. % were measured for **C+BAPE** and **C+DETA** as well.

In terms of CO_2_ uptake improvement, the prepared activated carbons can be ranked in the following order: **C+MELA** (32.30 wt. %) > **C+BAPE** (23.46 wt. %) > **C+DETA** (19.28 wt. %) > **C+MELA (P)** (18.31 wt. %) > **C+BAPE (P)** (9.64 wt. %) > **C+DETA (P)** (7.75 wt. %). In the hydrothermal and thermal samples (see Table 4), the following trend among the two carbons can be observed: the adsorption capacity increases with the increasing number of amine groups (nitrogen atoms) in the formula of amine used as an in situ *N*-modifying agent. This proves that the CO_2_ uptake is dependent on the basicity of the materials, which significantly contributes to the polarisation of the non-polar (but with quadrupole moment 14.3 × 10^40^ C m^2^) and slightly acidic CO_2_ molecules [58]. This was further confirmed by adsorption measurements of carbon dioxide on hydrothermal (**C**) and pyrolysed (**C (P)**) cellulose, for which the resulting CO_2_ adsorption capacities were 0.17 wt. % and 0.23 wt. %, respectively, at 0 °C and 1 bar (see Figure 8 and Table 4). The opposite trend was observed for these materials; thus, the pyrolysed material showed better CO_2_ adsorption properties than the hydrothermal product. This observation can be explained by the absence of primary absorption sites for CO_2_ in the form of amine groups. In such materials, an almost linear relationship between the specific surface area (*S_BET_*) and CO_2_ adsorption capacity is often observed, i.e., the amount of CO_2_ adsorbed increases as the *S_BET_* value increases [59].

**Table 5:** Summary of CO_2_ adsorption capacity measured on selected biomass-derived APCs at 0 °C and 1 bar.

Precursor of APCs	Activation	Activating Agent	Activation Method	*S_BET_* (m^2^ g^−1^)	Adsorption Capacity at 0 °C and 1 Bar (mmol)	References
Peanut shell char	chemical	KOH	homogenisation	1713	7.25	[60]
Black locust	chemical	KOH	impregnation	2064	7.19	[61]
Fern Leaves	chemical	KOH/CO_2_	impregnation	937	6.77	[62]
Pine Cone	chemical	KOH	impregnation	1787	6.57	[63]
Arundo donax	chemical	KOH	homogenisation	1122	6.30	[64]
Pomegranate peels	chemical	KOH	impregnation	585	6.03	[65]
Rice husk	chemical	KOH	homogenisation	1486	5.83	[66]
Coconut Shell	chemical	CO_2_	impregnation	1327	5.60	[67]
Sugarcane bagasse	chemical	LiOH	homogenisation	1149	5.50	[68]
Palm sheath	chemical	KOH	homogenisation	1052	5.28	[69]

**Figure 9:**
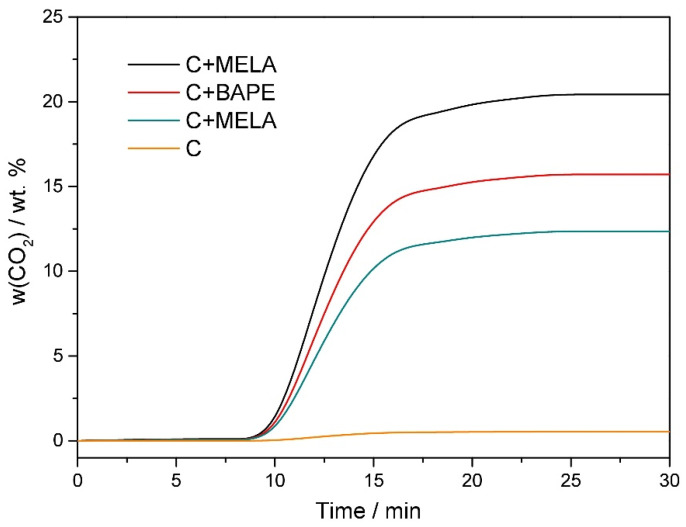
Thermogravimetric carbon dioxide adsorption curves of **C+MELA** (black curve), **C+BAPE** (red curve), **C+DETA** (green curve), and **C** (orange curve) measured at 25 °C.

In addition to the volumetric method described above, the CO_2_ capture ability of the materials was measured by the gravimetric method at 25 °C, with the obtained results shown in Figure 9. Only hydrothermal samples were subjected to these measurements, as they displayed higher CO_2_ adsorption capacity compared to the thermal materials. The hydrothermal APCs were initially thermally activated at 110 °C under an inert argon atmosphere and subsequently cooled to 25 °C. Upon introducing CO_2_ gas flow (20 cm^3^ min^−1^) after 7 min of the experiment, a rapid mass increase was observed due to the capture of CO_2_ molecules by the amine active sites of the modified samples or their pores. The total amount of adsorbed CO_2_ was 20.43 wt. % for C+MELA, 15.72 wt. % for C+BAPE, 12.36 wt. % for C+DETA, and 0.64 wt. % for C. It should be noted that the obtained values from thermogravimetric CO_2_ adsorption are lower compared to the values from the volumetric method. This can be attributed to the temperature difference under which experiments proceeded (0 °C for the volumetric and 25 °C for the gravimetric methods). Because the adsorption phenomenon is an exothermic process, the amount of adsorbed gas generally decreases with increasing temperature. The temperature of 25 °C was intentionally chosen to test the material’s applicability for CO_2_ capture under standard and real conditions.

As seen in Figure 9, a significant mass increase in the samples could be observed in the 7–15 min range upon exposure to CO_2_ gas, suggesting successful adherence of CO_2_ molecules to the surface of APCs. The increase in weight occurs from the seventh minute of the experiment because in the time interval 0–7 min the flow of argon was turned on, then the supply of Ar was turned off and the flow of gaseous carbon dioxide was turned on (time interval 7–30 min). It is possible to observe from the thermogravimetric adsorption curves that equilibrium was reached after 15 min of constant CO_2_ flow, as indicated by the flat plateau.

### 2.6. Recyclability

**Figure 10:**
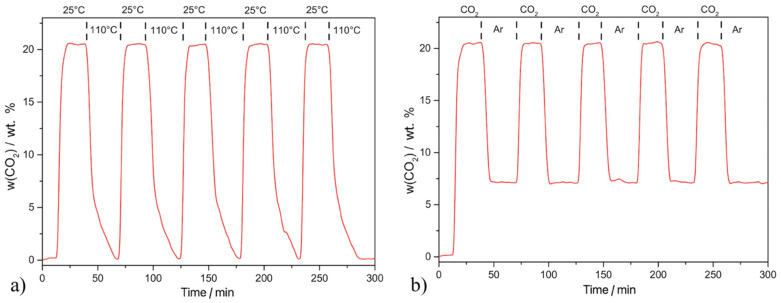
Cyclic thermogravimetric CO_2_ adsorption/desorption curve on **C+MELA** measured at 25 °C via (**a**) thermal regeneration at 110 °C and (**b**) argon purging at 25 °C.

The recyclability of **C+MELA**, the material which displayed the highest carbon dioxide adsorption capacity of 32.3 wt. % at 0 °C and 1 bar and 20.43 wt. % at 25 °C, was investigated via thermal regeneration at 110 °C (Figure 10a) and argon purging (Figure 10b). The sample showed high recyclability in both cases and retained a constant adsorption capacity (20.43 wt. %) after five adsorption/desorption cycles.

The material was regenerated entirely through thermal regeneration at 110 °C, as indicated by a mass residue of zero on the TG curve (see Figure 10a). In the case of argon purging realised at 25 °C, the compound showed high retainability leading to only partial regeneration of the sample, as indicated by a partial residue (~7 wt. %) in the desorption process (see Figure 10b). This can be attributed to the formation of carbamates caused by the reactions between the primary/secondary amines on the surface of the APC and the CO_2_ molecules, resulting in the formation of covalent bonds (chemisorption).

The sorption process can be divided into physisorption and chemisorption. During physisorption, adsorbates interact with the surface of adsorbent through weaker intermolecular interactions. During chemisorption, the adsorbate chemically reacts with the adsorbent to form a strong covalent bond. In **C+MELA**, both processes proceed simultaneously. Chemisorption of CO_2_ took place on amine groups under formation of carbamates, whereas physisorption occurred on the sample’s porous surface (1079 m^2^ g^−1^). Thermal regeneration at 110 °C managed to both completely remove the physisorbed/chemisorbed CO_2_ molecules from the pores and decompose carbamates, resulting in complete desorption of CO_2_. On the other hand, argon purging did not provide sufficiently strong energy flow to break the covalent bonds in carbamates. However, though insufficient to remove chemisorbed CO_2_ molecules, it was able to remove the physisorbed CO_2_ from the pores. For this reason, the TG curve in Figure 10b shows a partial residue (approx. 7 wt. %), which can be attributed to the mass of chemically bonded CO_2_ to melamine molecules within the **C+MELA** framework.

## 3. Experimental

### 3.1. Materials

Microcrystalline cellulose (99% pure; Central Chem, Charlotte, NA, USA) was used as the primary precursor for the synthesis of activated porous carbons. Diethylenetriamine (99% pure; Fisher Scientific, Waltham, MA, USA), 1,2-bis(3-aminopropylamino)ethane (94% pure; Sigma Aldrich, St. Louis, MO, USA), and melamine (99% pure; Sigma Aldrich) were employed as *N*-doping modifiers. KOH pellets (95% pure), KBr (≥99%; Sigma Aldrich), and HCl solution (35%; Central Chem) were used during the chemical activation.

### 3.2. Preparation of Hydrothermal Activated Carbons

**Figure 11:**
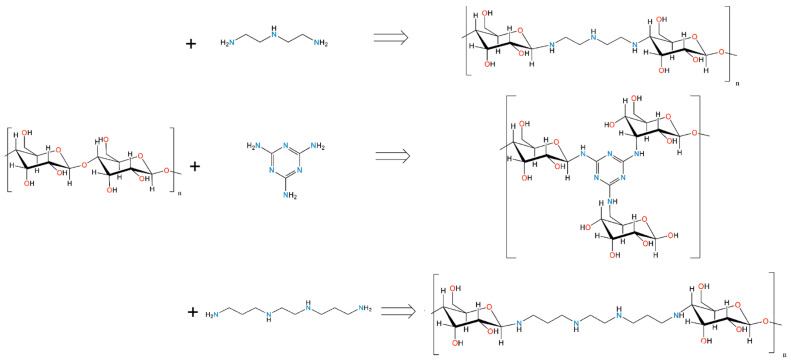
Scheme for preparation of nitrogen-doped cellulose–amine activated porous carbons.

Figure 11 illustrates the synthesis of cellulose–amine porous materials. Hydrothermal samples (see Figure 1, up) were prepared by mixing 1.3 g (8.02 mmol) of microcrystalline cellulose and 1.3 g of certain amines (diethylenetriamine (DETA) 12.60 mmol, 1,2-bis(3-aminopropylamino)ethane (BAPE) 7.46 mmol, and melamine (MELA) 10.31 mmol), which were placed in lined steel Parr autoclaves with 45 mL volume. Then, 27 mL of deionised water (DI) was added to the autoclaves using a pipette. The reaction vessels were sealed and placed into an oven to undergo hydrothermal activation at 240 °C for 8 h at a heating rate of 3 °C min^−1^ with subsequent cooling at 1 °C min^−1^. After cooling, the suspensions were washed with DI water using the Büchner funnel and dried at 120 °C in the oven overnight. The resulting hydrothermal products were denoted as **C+DETA**, **C+BAPE**, and **C+MELA**, yielding 0.49 g, 0.86 g, and 1.03 g, respectively.

### 3.3. Preparation of Pyrolysed Activated Carbons

The synthesis of thermal products consisted of the same initial hydrothermal activation and drying process as described above, followed by KOH chemical activation and pyrolysis (see Figure 1 down). For each sample, 0.9 g of potassium hydroxide was transferred to a pestle and mortar, where it was manually homogenised and mixed with 0.3 g of hydrothermal product in a 3:1 mass ratio. The prepared mixture was then placed into ceramic crucibles and transferred to an insulated tube furnace (Nabertherm), where the mixtures were pyrolysed under an inert gaseous environment (Ar) at 800 °C and heating rate of 3 °C min^−1^. This temperature was maintained for 1 h, after which the samples were cooled to the ambient temperature. After thermo-chemical treatment, the materials were washed with DI water multiple times to remove KOH residue and then mixed with diluted HCl solution. This purification process consisted of the following steps. Initially, the prepared materials were mixed with 40 mL of DI water, transferred to a plastic vial, and placed in the centrifuge. The liquid and solid phases of the suspension were separated at 3500 rpm for 10 min. Subsequently, the materials were mixed with 40 mL of deionised water and neutralised with a small amount of HCl solution until a neutral pH was attained. Solid materials were filtered using a Büchner funnel and dried at 80 °C for 18 h. The prepared products were ground and denoted in the study as **C+DETA (P)**, **C+BAPE (P)**, **C+MELA (P)**.

### 3.4. Characterisation of Activated Porous Carbons

The chemical composition of the compounds was determined using a CHN/O elemental analyser (Vario MICRO, Elemental Analysensysteme GmbH, Langenselbold, Germany). The oxygen content was calculated for each sample as the difference between 100% and the weight percentage sum of C, H, and N elements.

The chemical composition of the prepared APCs was studied by infrared spectroscopy at room temperature after gradually heating the compounds to selected temperatures (50, 100, 150, 200, 250, 300, 350, 400, 450, 500, 550, 600, 650 and 700 °C) using an Avatar 6700 FT-IR spectrometer in the range of wavenumbers 4000–400 cm^−1^. The samples were prepared as KBr tablets with a mass ratio of 1/100 (compound/KBr) and measured using the transmission technique. Before measurements were taken, KBr was dried at 700 °C to remove water moisture from the compound. KBr tablets were heated to the selected temperatures for 10 min for heating IR spectroscopy, then cooled to ambient temperature and measured. For each spectrum, 32 repetitions of scans were recorded with a resolution of 4 cm^−1^. The infrared spectra of the materials at room temperature were measured on the same instrument using the ATR (attenuated total reflectance) technique by the method of reflection on a diamond crystal. For each spectrum, 64 repetitions of scans were recorded with a resolution of 4 cm^−1^.

Thermal stability was investigated by thermogravimetry (TG) in an air atmosphere (60 cm^3^ min^−1^) with a heating rate of 10 °C min^−1^ and in a temperature range of 20–700 °C. Prior to the measurements, the samples were manually homogenised using pestle and mortar and then transferred to corundum (Al_2_O_3_) crucibles. TG curves were measured with a STA 449 F3 Jupiter Thermal Analyzer (Netzsch, Germany). The weights of the analysed samples were in the range of 10–20 mg.

Morphological aspects of the prepared products were studied using a field emission scanning electron microscope (JEOL JSM-7000F, Peabody, MA, USA). The powdered samples were compressed into cylindrical tablets and attached to the metal holder. The materials were then inserted into a microscope and evacuated. Subsequently, images of the materials with a magnification of 2500 were collected.

Textural properties of the prepared APCs were investigated by nitrogen adsorption/desorption measurements at −196 °C on an ASAP 2020 Plus analyser (Micromeritics, GA, USA) within a N_2_ relative pressure (*p*/*p*_0_) range of 10^−5^—1. Prior to the experiments, the samples were outgassed for 12 h under vacuum at 150 °C. Their porosity was evaluated by obtaining the Brunauer–Emmett–Teller specific surface area (*S_BET_*) [70] and respecting Rouquerols’s consistency criteria [55,71]. Pore size distributions were calculated by fitting the experimental adsorption data with the NLDFT (Non-Local Density Functional Theory) adsorption kernel.

The CO_2_ uptake of the prepared materials was evaluated on an ASAP 2020 Plus analyser (Micromeritics) and a STA 449 F3 Jupiter Thermal Analyzer via volumetric and thermogravimetric analysis at 0 and 25 °C, respectively, using high-purity CO_2_ (99.9995%) purchased from the Linde Gas Company. Degassing of materials and measurement of CO_2_ adsorption by the volumetric method was performed as described above for N_2_ adsorption/desorption measurements. After volumetric measurements, thermogravimetric experiments were carried out with the same already degassed samples; therefore, a lower activation temperature was applied. Prior to thermogravimetric measurements, the samples were thermally activated at 110 °C and subsequently cooled under argon atmosphere to 25 °C. During the thermogravimetric measurements, the flow rate of CO_2_ and Ar was 20 cm^−3^ min^−1^. The setup of the experiment was as follows: in the time interval 0–7 min, the flow of argon was turned on; in seventh minute, the supply of Ar was turned off; and in the time interval 7–30 min, the flow of carbon dioxide was turned on.

The recyclability of the sample with the highest CO_2_ adsorption capacity (**C+MELA**) was investigated in five adsorption/desorption measurement cycles using argon purging and thermal regeneration. In thermal regeneration, the sample was first saturated with pure CO_2_ gas (20 cm^−3^ min^−1^) during the adsorption process; then, during the desorption, the material was thermally activated at 110 °C. Because thermal regeneration is considered energetically demanding, the material’s recyclability was investigated using argon purging (20 cm^−3^ min^−1^), during which the sample was exposed to the flow of pure CO_2_ gas during adsorption and subsequently purged with Ar gas during the desorption process.

## 4. Conclusions

In this work, the effect of the activation method on the development of the physicochemical properties and CO_2_ adsorption capacity of activated porous carbons was investigated. It was found that while thermal activation led to significant enhancement of the textural properties of the materials, high activation temperatures applied during pyrolysis resulted in a notable decrease in the content of amines (nitrogen), which in turn led to weakened affinity for CO_2_ molecules, resulting in lower adsorption capacity. On the other hand, one-step hydrothermal activation resulted in the development of highly porous products with significantly greater CO_2_ adsorption capacities at temperatures of both 0 and 25 °C. Therefore, it can be concluded that hydrothermal activation represents a promising approach to addressing the environmental and economic impacts associated with the synthesis of biomass-based porous materials for CO_2_ capture.

The highest CO_2_ adsorption capacity of 32.3 wt. % was reported on melamine-containing activated carbon (**C+MELA**) at 0 °C and 1 bar. In addition to effective capturing ability, this sample displayed a well developed porous matrix, low production cost, and high recyclability, which in the case of application for large-scale CO_2_ capture can significantly reduce industrial expenses related to material purchase and waste regulation.

In summary, the application of biomass cellulose precursor and *N*-doping modifiers with high nitrogen content enabled the preparation of APC sorbents with remarkable CO_2_-capturing ability and recyclability. The newly developed **C+MELA** APC synthesised through a facile one-step hydrothermal method outperformed many of the high-performing activated-carbon sorbents reported in the literature in terms of selective CO_2_ capture. Additionally, this sample displayed a high level of sustainability and regenerability, which can significantly lower the cost of the post-combustion capture process. Therefore, this study presents a new approach to the preparation of solid-based CO_2_ capture materials and demonstrates that the proper selection of precursor, modifiers, activation method, and synthesis conditions is vital for the development of effective, low-cost, and recyclable CO_2_ adsorbents.

## Figures and Tables

**Table 1:** Results of elemental analysis (C, H, N, O in wt. %) for hydrothermal and thermal products.

	*N*_N_ *	C/%	H/%	N/%	O **/%
**C+DETA**	3	41.05	7.67	6.53	44.75
**C+BAPE**	4	43.69	8.18	7.84	40.29
**C+MELA**	6	37.84	6.35	12.61	43.20
**C+DETA (P)**	3	88.56	0.96	0.27	10.21
**C+BAPE (P)**	4	91.15	1.15	0.42	7.28
**C+MELA (P)**	6	84.98	0.73	0.67	13.62

*—number of nitrogen atoms in used amines (see Figure 2), **—the content of O was calculated as the difference between 100% and the weight percentage sum of C, H, and N elements.

**Table 2:** Wavenumbers (in cm^−1^) of relevant adsorption bands in the IR spectra of prepared APCs.

	C	MELA	DETA	BAPE	C+MELA	C+DETA	C+BAPE	C+MELA (P)	C+DETA (P)	C+BAPE (P)
ν(OH)	3332 (as) 3281 (s)	X	X	X	X	X	3403	X	X	X
ν(NH_2_)	X	3467 3417	3353 3276	3353 3276	3468 3412	3351 3276	3331 3284	X	X	X
ν(NH)	X	X	3184	3176	3328	3184	3180	X	X	X
	X	3176 3120	X	X	3184 3130	X	X	X	X	X
ν(CH_2_)_aliph_	2895	X	2925, 2879 (as) 2854, 2812 (s)	2922(as) 2813(s)	2962, 2931 (as) 2898, 2868 (s)	2925, 2876 (as) 2851, 2812 (s)	2915 (as) 2848, 2823 (s)	X	X	X
δ(OH)	1645	X	X	X	X	X	X	X	X	X
δ(NH_2_)	X	1626	1595	1598	1649 1624	1595	1590	X	X	X
ν(CN)	X	1566 1526	X	X	1552	X	X	X	X	X
δ(CH_2_)	1428	X	1454	1456	1435	1454	1455	X	X	X
δ(CH)	1333	X	1351	1356	1363	1351	1358	X	X	X
δ(C-OH)	1314	X	X	X	1313	1300	1314	X	X	X
ν(C-O)	1053 1028	X	X	X	1052 1022	1062 1036	1055 1031	X	X	X
γ(CCH)	X	X	X	X	X	X	X	991	977	992

aliph—aliphatic, s—symmetric, as—asymmetric, X–not present.

**Table 3:** Textural properties (BET surface area (*S_BET_*) and pore volume (*V_p_*)) of activated hydrothermal and pyrolysed carbons and of carbonaceous materials modified with different amines.

	*S_BET_* m^2^ g^−1^	*V_p_* cm^3^ g^−1^
**C**	32	0.014
**C+DETA**	769	0.378
**C+BAPE**	833	0.473
**C+MELA**	1079	0.511
**C (P)**	121	0.067
**C+DETA (P)**	998	0.488
**C+BAPE (P)**	1076	0.502
**C+MELA (P)**	1348	0.628

**Table 4:** CO_2_ adsorption capacity of APCs at 0 °C and 1 bar in terms of volume (*V*), amount of substance (*n*), and mass fraction (*w*).

	*V*(CO_2_)cm^3^ g^−1^	*n*(CO_2_)mmol g^−1^	*w*(CO_2_)wt. %
**C**	3.65	0.17	0.75
**C+DETA**	98.08	4.38	19.28
**C+BAPE**	119.35	5.33	23.46
**C+MELA**	164.60	7.34	32.30
**C (P)**	5.18	0.23	1.01
**C+DETA (P)**	39.54	1.76	7.75
**C+BAPE (P)**	49.04	2.19	9.64
**C+MELA (P)**	93.29	4.16	18.31

## Data Availability

The data presented in this study are available in article and Supplementary Materials.

## Supplementary Materials

The following supporting information can be downloaded at: https://www.mdpi.com/article/10.3390/molecules29051158/s1, Table S1: The identification of notable mass decreases of the hydrothermal APCs in particular temperature intervals.
