# Supplementary material for: Cellulose–Amine Porous Materials: The Effect of Activation Method on Structure, Textural Properties, CO2 Capture, and Recyclability
Source: Molecules. 2024 Mar 5;29(5):1158. doi: 10.3390/molecules29051158 (PMC10934251; doi:10.3390/molecules29051158)
Supplement: Supplementary file 1 [file molecules-29-01158-s001.zip › molecules-2884516-SI.pdf]

Table S1. The identification of notable mass decreases of the hydrothermal APCs in particular temperature intervals.

		Water			Amine		Cellulose	
	**Thermal stability**	**Temperature interval 1. / °C**	**Mass decrease / wt. %**	**Stability after dehydration**	**Temperatur e interval 2. / °C**	**Mass decrease / wt. %**	**Temperatur e interval 3. / °C**	**Mass decrease / wt. %**
**C**	58.8	58.8-136.9	7.6	136.9-288.0	X	X	288.0-511.0	92.4
**C+DETA**	43.5	43.5-115.2	5.0	115.2-265.3	265.3-332.3	22.1	332.3-508.9	72.9
**C+BAPE**	53.8	53.8-134.1	8.5	134.1-254.8	254.8-341.4	25.4	341.4-542.2	66.1
**C+MELA**	39.9	39.9-163.3	11.1	163.3-315.7	315.7-380.1	20.4	380.1-517.4	68.5
**C+DETA (P)**	X	X	X	115.2-265.3	265.3-332.3	22.7	332.3-508.9	77.3
**C+BAPE (P)**	X	X	X	134.1-254.8	254.8-341.4	27.1	341.4-542.2	72.9
**C+MELA (P)**	X	X	X	163.3-315.7	315.7-380.1	22.7	380.1-517.4	77.3
